# Biomimetic Silica Microspheres in Biosensing

**DOI:** 10.3390/molecules15031932

**Published:** 2010-03-17

**Authors:** Sireesha Chemburu, Kyle Fenton, Gabriel P. Lopez, Reema Zeineldin

**Affiliations:** 1 Center for Biomedical Engineering, University of New Mexico, Albuquerque, NM 87131, USA; 2 Department of Biomedical Engineering, Duke University, Durham, NC 27708, USA; 3 Department of Mechanical Engineering & Materials Science, Duke University, Durham, NC 27708, USA; 4 Department of Pharmaceutical Sciences, Massachusetts College of Pharmacy and Health Sciences, 19 Foster Street, Worcester, MA 01608, USA

**Keywords:** biomimetic, silica, microsphere, porous, supported lipid bilayer, fluorescence, flow cytometry, microfluidic, biomembrane

## Abstract

Lipid vesicles spontaneously fuse and assemble into a lipid bilayer on planar or spherical silica surfaces and other substrates. The supported lipid bilayers (SLBs) maintain characteristics of biological membranes, and are thus considered to be biomembrane mimetic systems that are stable because of the underlying substrate. Examples of their shared characteristics with biomembranes include lateral fluidity, barrier formation to ions and molecules, and their ability to incorporate membrane proteins into them. Biomimetic silica microspheres consisting of SLBs on solid or porous silica microspheres have been utilized for different biosensing applications. The advantages of such biomimetic microspheres for biosensing include their increased surface area to volume ratio which improves the detection limits of analytes, and their amenability for miniaturization, multiplexing and high throughput screening. This review presents examples and formats of using such biomimetic solid or porous silica microspheres in biosensing.

## 1. Introduction

Supported lipid bilayers (SLBs) on microspheres serve as a biomimetic platform for several biotechnology applications such as biosensing, molecular interactions, and drug delivery [1,2,3,4,5,6,7,8,9,10,11,12,13,14]. Lipid bilayers are made of two leaflets; each comprises a layer that is made of amphiphilic phospholipids with hydrophobic tails of each leaflet oriented inwards to face each other, while the polar heads are oriented outwards towards aqueous environments. This structure of the lipid bilayers mimics biological membranes but it represents a very simplified model as it does not contain any membrane proteins or specialized membrane microdomains such as lipid rafts. On the other hand, integral membrane proteins will go into lipid bilayers with maintaining their structure and functionality due to their assumption of active conformations within the lipid bilayers. Furthermore, non-integral membrane proteins can be incorporated into lipid bilayers through lipid anchors while maintaining their functionality. Another way in which lipid bilayers mimic the properties of biological membranes is that they serve as barriers to ions and hydrophilic molecules. 

SLBs have been formed on spherical or flat substrates made of different materials including silica, glass, mica or polymeric supports [12,15,16]. Lipid vesicles are used to form SLBs as they spontaneously fuse and assemble into a lipid bilayer on substrates [15]. An SLB on a microsphere mimics a cell-like structure that is covered with a biomembrane-mimetic lipid bilayer, and is thus referred to as biomimetic microsphere in this review. SLBs have a thickness of 5 ± 1 nm with a 1.2–2.2 nm water interface between the SLB and the underlying spherical substrate [17]. In SLBs the polar heads in the upper leaflet face the exterior solution whereas the ones in the lower leaflet face the supporting substrate [15]. SLBs, just like lipid bilayers within vesicles maintain lateral fluidity, surface motility, and self-healing properties [6,15]. The main limitation of SLBs is their proximity to the underlying substrates causing possible interaction of integral proteins within them with the substrate. Ways to decouple the SLBs from substrate influences that were successfully employed with planar supports include introducing spacers such as tethering, using a polymer cushion, or using patterned surfaces to suspend the lipid bilayer on [15,16,18,19,20,21]. Tethering of SLBs on microspheres was also demonstrated [22,23].

This review will present the use of biomimetic silica microspheres in biosensing with spheres ranging in size from submicrons to several microns. Although microspheres other than silica have been used to form SLBs, they are outside the scope of this review and are reviewed by Troutier and Ladaviere [12]. In the current review we first define biosensors, and then we describe methods for preparing biomimetic silica microspheres, and present their characteristics, detection methods, biosensing formats, and end by listing biosensing applications that have used biomimetic silica microspheres so far.

## 2. Biosensing

**Figure 1:**
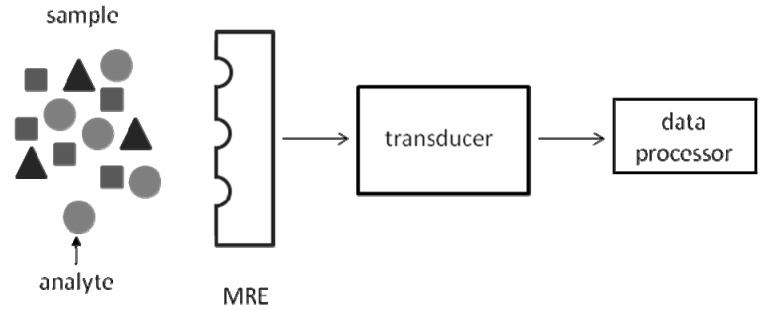
Fundamental components of a biosensor. A biosensor consists of a molecular recognition element (MRE), a physical transducer and a data processor.

In general, a biosensor consists of a molecular recognition element (MRE) and a physical transducer [24], where upon binding of a target bioanalyte to the MRE, a change in a physical parameter occurs and the transducer serves to convert this detected change into an electrical or digital signal (Figure 1). The MRE imparts specificity and it may be a protein (receptor, antibody, enzyme, surface protein, ion channel), or an oligonucleotide. In addition, artificial materials may be utilized as MREs including aptamers, peptide nucleic acids (PNAs) or molecularly imprinted polymers (MIPs). The extent of a biorecognition event is reflected in a measurable change in a physical quantity such as an optical, electrochemical, thermal, or magnetic property which can be detected by the transducer [24]. 

## 3. SLBs on Silica Microspheres

Biomimetic microspheres are generated by supporting lipid bilayers on microspheres. Lipid bilayers or even monolayers are formed on microspheres by various methods, including adsorption, and chemical crosslinking [6,17,22,25]. Different types of microspheres are used in biosensing and are available in a wide range of sizes including polymeric, magnetic, silica, or even biological particles [12,26]. The bulk work of SLBs on microspheres used in biosensing has employed silica microspheres, and thus this review focuses on biomimetic silica microspheres and their use in biosensing. 

**Figure 2:**
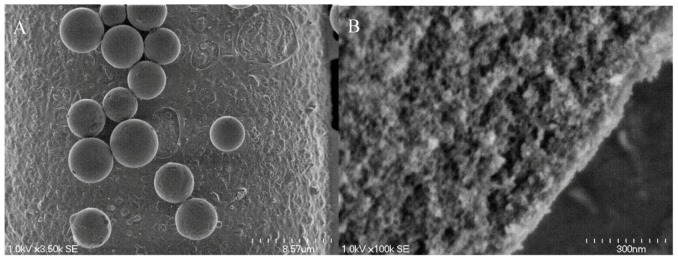
Images of solid and porous microspheres. (A) Scanning EM image of a non-porous microsphere with an average diameter of ~ 5 μm, and (B) high resolution transmission EM image of a porous microsphere showing the porous surface.

Silica microspheres have many properties that make them attractive for use in biosensing. These properties include being chemically inert, having low autofluorescence, and their surface can be easily functionalized using organosilanes or chemical conjugations to biomolecules. In addition to solid silica microspheres, mesoporous silica microspheres with controllable pore sizes and ordering have been customized to become suitable in sensing applications [27,28,29,30,31,32]. Figure 2 shows electron microscopy (EM) images of solid (non-porous) and porous silica microspheres. Mesoporous silica microspheres exhibit uniform pore sizing and ordering while providing a high surface area to volume ratio. Their pore properties provide a good way to deliver and store materials like fluorophores or biomolecules that exist intracellulary, thus creating a cell-like environment. Both porous and solid microspheres have been used in biosensing in the form of biomimetic microspheres. To generate biomimetic silica microspheres, liposomes in the form of unilamellar vesicles are first prepared and then they are used to coat silica microspheres. In addition, other natural membrane fragments or cell-derived vesicles can also be integrated into liposomes or used directly to form SLBs on microspheres.

## 4. Materials Used in SLBs

### 4.1. Liposomes

**Figure 3:**
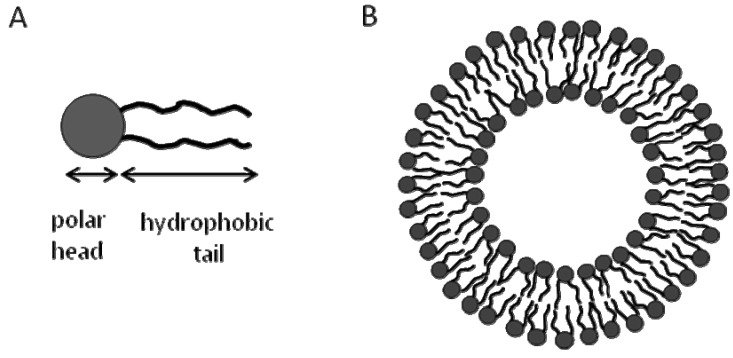
Schematic of a liposome. (A) A schematic of a phospholipid showing the hydrophilic and hydrophobic parts. (B) A cross section in a unilamellar liposome vesicle showing the two leaflets of the lipid bilayer which has a thickness of 5 nm.

Liposomes are made of phospholipid molecules that spontaneously self-assemble into spherically closed bilayer membranes [33,34,35], where the polar heads are hydrated with water, whereas the hydrophobic hydrocarbon chains of each leaflet of the bilayer core interact with each other (Figure 3). The thickness of the lipid bilayer of the liposomes is typically about 5 nm [33]. Typical phospholipids used to prepare liposomes include phosphatidyl cholines (PC), phosphatidyl ethanolamines (PE), and phosphatidyl glycerols (PG) [33,34,35]. In addition, other lipids are usually incorporated into liposomes such as cholesterol, phosphatidyl inositol (PI), phosphatidyl serine (PS), and sphingomylin [33,34,35]. The phospholipid composition of liposomes defines membrane surface properties; for example interaction with charged supports, interaction with membrane proteins, permeability and wetting are highly dependent on presence of charged lipids [36]. Liposomes, whether unilamellar or multilamellar, are prepared by a variety of methods at a temperature above their gel-to-fluid transition temperature (Tm), and they vary in size from tens of nanometers to tens of micrometers, thus forming nano- or micro-spheres [33,37].

Liposomes mimic cell membranes as they form a lipid bilayer into which membrane proteins could be inserted. The advantages of using liposomes as membrane mimics are that they are fluid, thus allowing lateral mobility, and self-healing [33,34,35]. The self-healing properties result from the free diffusion of lipids within the two leaflets of the lipid bilayer. Furthermore, liposomes are impermeable to ions, and they suppress non-specific binding of proteins. Liposomes have been used as an embedding medium for incorporating transmembrane proteins, such as pore-forming proteins and ion channels. In addition, the surface of liposomes may be modified by attaching biomolecules *via* covalent binding or using biotin-avidin coupling [6,38,39,40]. Using liposomes as biomimetic microspheres has been problematic due to their instability. However, this was easily overcome by supporting lipid bilayers or even monolayers on microspheres. This is accomplished by adsorption or by covalent modification of the sphere surface as described below under section 5. In conclusion the above properties of lipid bilayers within liposomes and the ability to incorporate membrane proteins make them appealing biomembrane mimetic systems that can be further stabilized by adsorbing onto microspheres for use in biosensing applications.

### 4.2. Cell-derived components

Components derived from mammalian or bacterial cells have been employed with or without SLBs in forming SLBs on substrates. Examples of mammalian cell-derived components include proteoliposomes of mitochondrial inner membranes [41], and erythrocytes’ membranes [42]. Examples of bacterial-derived components include bacteriorhodopsin (bR) [7,22], and *E. Coli* inner membranes [43]. Both mammalian- and bacterial-cell derived examples are presented here along with their advantages and disadvantages.

#### 4.2.1. Mammalian cell-derived components

Erythrocytes’ ghost membranes prepared by hypotonic lysis were successfully deposited on solid and porous silica microspheres [42]. Nevertheless, they had an inverted orientation with exposing the cytoplasmic face to the outside environment [42]. These membranes were intact and proteins within them maintained their functionality [42]. However, depositing these membranes with the right side out would be beneficial in mimicking the cell to study interactions of some membrane proteins with intracellular molecules that can be stored within porous silica microspheres, but preparation with the right side out may present a challenge. 

Preparation of proteoliposomes of mitochondrial inner membranes involves isolating inner mitochondrial membranes that are then mixed and sonicated with lipid vesicles to produce proteoliposomes with controlled lipid to protein ratio [41]. These were used to form SLBs on flat substrates which involved multiple steps; starting with tethering of the proteoliposomes by biotin/streptavidin interactions on a flat substrate coated with polyethylene glycol to prevent nonspecific interactions [41]. This was followed by triggering fusion of the proteoliposomes resulting in formation of solid-supported mitochondrial membranes [41]. Although these proteoliposomes were used to form solid-supported mitochondrial membranes on flat substrates, they can easily be supported on spherical substrates.

#### 4.2.2. Bacterial-derived components

In addition to utilizing mammalian cell-derived components to form SLBs on substrates, bacterial-derived components are also employed. For example, purple membranes containing bacetriorhodopsin (bR) have been integrated into SLBs on silica microspheres [7,22], where even in one case bR was used to template the assembly of a suspended SLB on microspheres [22]. The advantage of purple membranes is that they are membrane fragments with limited size, so they are easily integrated into SLBs.

Another example of bacterial derived components that was assimilated into SLBs are *E. coli* inner membranes [43]. Proteoliposomes containing *E. coli* inner membranes were formed as described above for the mammalian-derived proteoliposomes of mitochondrial inner membranes, and then were supported on planar substrates. Although not reported yet, these can be easily supported on microspheres to create biomimetic microspheres. 

The advantage of mammalian or bacterial cell-derived vesicles is that the biorecognition element exists within physiological conditions, which help maintain their functionality. Furthermore, they contain additional cellular components which may be employed in biosensing or in investigating activation of the cell signal transduction pathways because they contain components of the membrane and the cytoplasm. Conversely, containing cellular components maybe a disadvantage; for example, if these components act as part of the cellular machinery for protein degradation then this may lead to limited stability of some proteins.

An important issue that needs to be tackled when using intact membranes in general is that these membranes are composed mostly of proteins which will hinder assimilation of biological membrane fragments into lipid bilayers or even formation of SLBs and will introduce rigidity. The alternatives are to use them as is, *i.e.* support the biological membranes directly on microspheres without mixing with SLBs, or incorporate them into lipid vesicles after disrupting internal interactions within the membranes possibly utilizing sonication, mild detergents or cholesterol depletion.

## 5. Preparation of SLBs on Silica Microspheres

**Figure 4:**
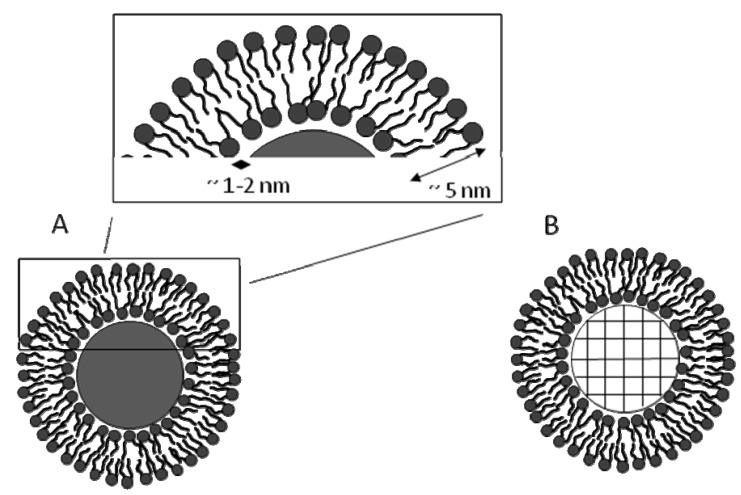
Schematic of a supported lipid bilayer (SLBs) which has a thickness of 5 nm with an underlying 1-2 nm water layer on (A) a solid microsphere, and (B) a porous microsphere.

Liposomes are deposited on solid microspheres by spontaneous adsorption, rupture and fusion to form SLBs on microspheres [6,17,44]. Typical SLBs are separated from the spherical substrate by a thin 1-2 nm water layer, and like natural membranes, they maintain their fluidity (Figure 4A) [17]. Furthermore, the same method is used to form SLBs on porous silica microspheres (Figure 4B) [6,7,45,46]. In the latter case, the connectivity of the internal pore space was confirmed by evaluating fluorescence recovery after photobleaching (FRAP) of an internal spot within fluorescent dye-encapsulated microspheres [6], and the diffusion constant was reported to be independent of the different sizes of microspheres [7]. The ordered pore network provides high surface area and pore volume, which was calculated to be ~4 orders of magnitude higher than surface area of equivalent size solid microspheres [7]. This enables these microspheres to serve as carriers of fluorescent dyes, biomolecules, or any other chemical agents that can be used in biosensing. 

Besides adsorption, other methods exist for preparation of more stable SLBs or supported lipid monolayers (SLMs) on microspheres. For example, phospholipids have been covalently linked to microspheres [47,48], and robust synthetic lipid coatings using alkoxysilane-based lipids were prepared using layer-by-layer assembly on polyelectrolyte-coated particles [49]. Bacteriorhodopsin (bR) has been employed in template-assembly of lipid bilayers by conjugating to bR biotinylated polyethylene glycol which bound to streptavidin-functionalized silica microspheres [50]. The bR serves to nucleate the self-assembling lipid bilayer around the silica microsphere. SLMs have been formed on silica microspheres through coating the microspheres with silanized hydrocarbon chains that form an interface with the lipid monolayer [25].

In addition to forming continuous SLBs on microspheres, it is possible to create membrane patterns on silica microspheres using *in situ* UV-ozone photolithography, which causes photochemical degradation of lipids in aqueous environment [51]. The patterning leads to formation of isolated zones on microspheres thus creating isolated areas on the microsphere surface that can serve as different reaction domains within each microsphere. To sum up, SLBs can be formed around microspheres either by physical adsorption or chemical conjugation to form continuous or patterned SLBs that are stable and can be used in various applications.

## 6. Characteristics of SLBs on Silica Microspheres

SLBs on microspheres have several advantages over liposomes, such as controllable shape and narrow size distribution, increased stability, ease of washing by centrifugation, and making microspheres biocompatible. When such biomimetic microspheres are used in biosensing applications, the SLB serves as an electric insulator, which is useful when combining biochemical processing with signal processing using hard components. Studying SLBs on microspheres involves methods different from those used with planar substrates. When lipid bilayers are supported on planar substrates, several techniques exist to characterize formation of SLBs on such substrates, including surface plasmon resonance (SPR), quartz crystal microbalance (QCM), microscopies, ellipsometry, and Fourier transform infrared spectroscopy (FTIR) [15,16,52,53,54,55]. However, with the exception of FITR and microscopic techniques [12], these techniques cannot be used to study SLBs on microspheres. This represents a deficiency with microspheres, and adaptations of similar techniques to study them are still lacking. In contrast, other techniques that are not feasible for planar SLBs are used to characterize the dynamics and structural properties of SLBs on microspheres including flow cytometry, nuclear magnetic resonance (NMR), and differential scanning calorimetry (DSC) [4,6,7,12,17,56].

It has been demonstrated that the SLB easily forms on a solid microsphere [17] or on a porous microsphere using unilamellar vesicles [6,7]. The SLBs on microspheres are impermeable to protons or dyes as confirmed by encapsulating into a porous microsphere a pH-sensitive dye, where the SLB-trapped dye did not leak out, nor did it change fluorescence upon changing the pH of the external environment [6]. The maintenance of fluid character of SLBs on microspheres was shown by DSC which was found to be comparable to multilamellar vesicles made of the same lipids without the microspheres [4]. Additionally, the fluidity and lateral diffusion were demonstrated by FRAP of a target region after incorporating a fluorophore-labeled phospholipid into the SLB [6,23]. Also lateral diffusion of phospholipids within the SLBs on microspheres is maintained as determined by deuterium NMR relaxation techniques [12]. Nevertheless, it seems that the lateral diffusion within the internal leaflet may be restrained by the substrate however that is dependent on the size and charge of the polar heads of phospholipids as detailed elsewhere [12]. 

The stability of SLBs on silica microspheres was dependent on the gel-to-fluid transition temperature (Tm) where lipids with lower Tm were more stable than ones with higher Tm [7]. In addition, the solid substrate appears to influence the Tm of the formed SLBs on microspheres by decreasing Tm by 2 °C in comparison to vesicles as evaluated by DSC [12,56]. In summary, it appears that the characteristics of lipid bilayers are maintained within SLBs with slight influence of the underlying substrate on the Tm and slowing lateral diffusion within the inner leaflet depending on lipids composition.

## 7. Incorporation of Functional Transmembrane Proteins into Biomimetic Microspheres

Several membrane proteins have been introduced into SLBs on microspheres with retaining their functions, which is important either for interaction with an analyte or for subsequent activation of elements that may be involved in the detection process. Examples of such proteins successfully used with biomimetic silica microspheres include bacteriorhodopsin (bR), which is a proton pump [7,22], human type 3 serotonin receptor (5HT3R), which is a receptor for neurotransmitters [7], ICAM-1 [6] and P-selectin [2], both are cell adhesion proteins, ganglioside GM1 which is a cell surface glycolipid [9], calcium-ATPase which transports calcium while hydrolyzing ATP [4], and cytochrome c oxidase which plays a role in electron transport [57].

Although these examples of integral membrane proteins retained their functions after being incorporated into SLBs on microspheres, this may not be the case for all integral membrane proteins. The close proximity of proteins with a cytoplasmic domain larger than few nanomometers to the substrate may inhibit lateral mobility and interfere with the functionality of the integral membrane protein. This problem may be more prominent with the solid microspheres rather than the porous ones, but the difference has not been explored yet. Measures decoupling the SLB from the substrate by using a cushioning polymer or using polymer tethers that increase the space between the lipid bilayer and the substrate have been successfully used with planar substrates [15,16,18,19,58] , and can be adapted to microspheres. A successful tethering of SLBs on microspheres was demonstrated by avidin-biotin interactions alone [23] or in addition to bR [22]. Further exploration of decoupling strategies that have been successful with planar substrates is yet to be performed with spherical substrates.

## 8. Detection Methods

Biosensing involves detecting a change in a physical quantity when a target analyte binds to the sensor system (Figure 1). In the case of biomimetic silica microspheres, this has been mainly in form of a change in fluorescence, which is detected using fluorimetry by a fluorimeter or a flow cytometer. Using these instruments, it is possible to detect an increase in fluorescence, quenching of fluorescence, or fluorescence resonance energy transfer (FRET), which are presented here. Additional methods of detection include radioactive methods and detection of transition from a microspheres’ condensed phase into a dispersed phase [5]. The latter is briefly presented under applications. The former was used with biomimetic polymeric microspheres to detect released radioactive fatty acids as a result of hydrolysis of phospholipids within SLBs on polymeric microspheres by the membrane active enzyme phospholipid A_2_ [59]. Fluorescent detection methods are attractive because they are less hazardous than radioactive ones and will be presented in this section.

### 8.1. Fluorimetry

Fluorescence detection techniques are the primary method used in biosensing. Fluorimetry relies on the excitation of a fluorophore and detection of its emission. In addition to detecting an increase in fluorescence, fluorimetry is used to detect reduction of fluorescence or quenching. Most typically used fluorimeters consist of monochromators, polarizers, filters and gratings and the detection is done using a photo-multiplier tube. All changes in fluorescence can be quantitated by fluorimetry or flow cytometry. 

Flow cytometry is a very sensitive method that permits simultaneous measurement of multiple fluorescence signals as a result of illumination of single cells or microscopic particles in suspension, as they flow rapidly through a laser beam. Furthermore, in flow cytometry inspection of heterogeneous populations is possible, which is hard to resolve using traditional methods such as fluorimetry [60]. The ability of a flow cytometry to measure between 5 and 10 distinct parameters per cell or particle (size, morphological features, and multiple fluorescent colors simultaneously) enables multiplexing and gating on homogeneous sub-populations within heterogeneous populations of cells or particles [61]. Cytometry even has the capability of employing standard fluorimetry techniques such as fluorescence resonance energy transfer (FRET) [62]. It has been used widely since the late seventies to determine many different reactions involving fluorescent probes such as antigen-antibody interactions, DNA hybridization and enzymatic reaction products. All of these have been successfully demonstrated on the surface of a microsphere. The inspection of a biomimetic microsphere is very advantageous using flow cytometry due to the ability to quantify several reactions at once provided the correct labels and reaction time. Another advantage of flow cytometry, besides multiplexing, is that it has recently become a high throughput technique as a result of the use of fast automated sample processing leading to rapid analysis of multiple samples and even using low volumes of samples [63].

#### 8.1.1. Fluorescence quenching

Fluorescence quenching is defined as a reduction in fluorescence of a fluorophore as a result of specific events such as energy transfer, excited state reactions, or collisional quenching. Detection of the release of self-quenching dyes from liposomes [64] or detection of the quenching of intrinsic protein fluorescence by quenchers [65] has been employed in biosensing using liposomes and is easily adaptable for use with biomimetic microspheres. In addition to using pairs of fluorophores and quenchers in biosensing, sensitivity is achievable by using fluorescent conjugated polymers [66]. Fluorescent conjugated polymers display high fluorescence quantum yields and enhanced sensitivity to quenching (superquenching) by oppositely charged quenchers through energy or electron transfer. Superquenching of fluorescent polymers adsorbed to or covalently attached onto solid supports such as functionalized polymeric particles have been used in immunological and kinase assays [67,68,69,70,71,72]. After being anchored or adsorbed onto polymeric microspheres, the fluorescent polymers retain or even increase their fluorescence intensity and their sensitivity to small molecule quenchers [14,73,74,75]. This also attenuates the non-specific adsorption of undesired charged biological molecules onto the fluorescent polymer [68].

#### 8.1.2. Forester or fluorescence resonance energy transfer (FRET)

Forester or fluorescence resonance energy transfer (FRET) is a non-radiative process in which the excitation energy from a donor fluorophore is transferred to an acceptor fluorophore by dipole-dipole interactions. The detection of FRET is a highly specific indicator of the proximity of the two molecules with separation distance of 1–10 nm [76,77]. Under steady state conditions, FRET is usually calculated by measuring emission, which involves the detection of the light emitted by either the donor and/or the acceptor in the presence of the other fluorophore. Other variations of FRET measure acceptor quenching [76] or acceptor photobleaching [77]. The reason that FRET has become a valuable tool for immunoassays is that the efficiency of energy transfer has a strong dependence on the distance between the donor and the acceptor. Thus to study interactions between two molecules, one is labeled with the donor fluorophore and the other with the acceptor fluorophore, and detection of FRET indicates interactions between the two fluorophore-labeled molecules.

## 9. Biosensing Formats Using Biomimetic Microspheres

**Figure 5:**
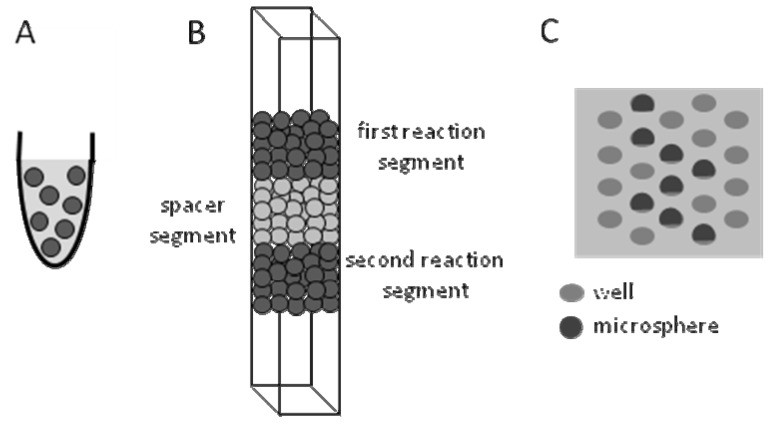
Platforms for using microspheres in biosensing include (A) in suspension in a tube, (B) packed in a channel (2 cm in length, 250 μm in width, and 60–70 μm in depth) that contains one or more reaction segments, and (C) within chemically-etched wells on an optical fiber where the microspheres fit into etched wells of nearly matching size.

The use of microspheres in biosensors has been carried out in various formats, in suspension, in packed channels or within chemically-etched wells on optical-fiber bundles (Figure 5) [6,14,45,78,79,80]. In comparison to planar formats, microspheres suspended in a tube (Figure 5A) have the advantage of providing better analyte transport to the reactive surfaces that can be driven, dependent on the type of microsphere, by applying electric fields, pressure, magnetic fields, or by simple shaking e.g. using a vortex [81]. Polydimethylsiloxane (PDMS) channels constructed using soft lithographic techniques have been packed with biomimetic microspheres for micofluidic biosensing applications [45]. Packed channels of microspheres while generating a pressure drop that may hinder convective mass transport have the benefit of significantly decreasing diffusive mass transport limitations because of their very high surface area. They also have the further advantage of allowing the introduction of multiple separated reaction segments within the channel (Figure 5B), where even sequentially dependent reactions may be used [11,45]. Chemically-etched wells on optical fibers can be customized with a specific size varying from few to tens of microns and have been loaded with molecules, functionalized microspheres, or live cells [79]. An optical fiber bundle has a diameter less that 1 mm and is made of fiber’s with a uniform diameter ranging between 2 to 20 μm [79]. Optical fiber bundles are useful in biosensing because they are tailored so that each fiber independently carries light over a long distance and fluorescent signals are observed simultaneously under a fluorescent microscope [79]. Microspheres placed within chemically-etched wells on optical-fiber bundles have the advantage of simultaneous detection of the microwells and this format has the flexibility of being mounted on a fluorescent microscope, which permits high throughput detection.

Lipid supported microspheres have been used so far in two formats; in suspension or packed in channels (Figure 5A, B). With biomimetic microspheres in suspension, fluorescence of a dye leaking into solution is easily detected after sedimenting the microspheres. Alternatively the fluorescence of the microsphere itself is detected using flow cytometry. In contrast, in the case of biomimetic microspheres packed into a channel, fluorescence as a result of dye release is detected at a point below the reaction segment that contains biomimetic microspheres. The channels are constructed using different materials including poly (dimethylsiloxane) (PDMS) and silicon [78,82,83]. To prevent microspheres from exiting the channel, frits, dams, or polymers inside of the microchannel have been used [84]. It was found that the SLBs of biomimetic microspheres packed into a channel are not disrupted by the packing process and thus can be employed in biosensing assays [45]. The advantage of this technique is that the close packing of biomimetic microspheres greatly increases the surface area to volume ratio. This increases the efficiency of the sensor by reducing the distance that an analyte must travel from the bulk fluid phase to the stationary phase. This packed channel technique can also be extended to handle multiplexed assays. The main drawback to this strategy is that as the amount of spheres’ packing increases, the flow rate typically decreases due to the high pressure loss that can occur across densely packed channels. This obviously depends on the microsphere size and packing length [11]. In conclusion, although biosensing microspheres has been used in three formats; in suspension, in packed channels or within chemically-etched wells on optical-fiber bundles, only the first two have been used with biomimetic microspheres, and some of the supplications they have been used in are presented in the next section.

## 10. Applications—Using Biomimetic Microspheres in Biosensing

**Figure 6:**
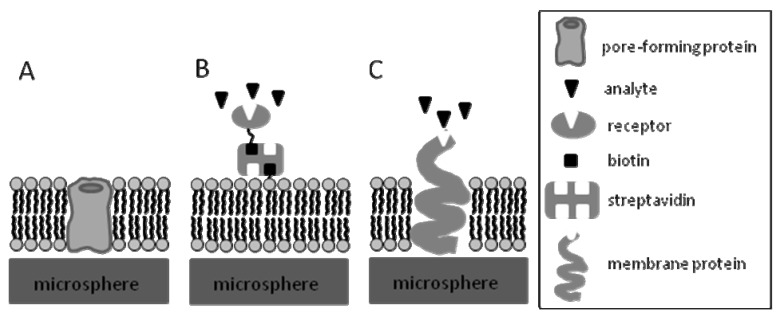
Schematic showing examples of interactions of biomolecules with SLBs. (A) Interaction with SLB by incorporating a pore-forming protein into the SLB. (B) Interaction of a ligand with a biomolecule tethered onto the SLB through biotin-streptavidin interactions. (C) Interaction of a ligand with a membrane protein.

Biosensing systems using SLBs on microspheres have been, so far, based on detecting changes in specific properties, including fluorescence or colloid-phase transitions, as a result of direct or indirect biointeractions of analytes with the lipid bilayer or with molecules within it. For example direct detection of membrane active peptides that either lyse the membrane or form a channel or a pore in it (Figure 6A), or detection of molecules that simply prefer partitioning into the lipid bilayer because of their lipophilicity. Alternatively, these systems can detect interactions of analytes with molecules incorporated into the bilayer or immobilized onto the bilayer *via* streptavidin-biotin coupling (Figure 6B) [6,38,39,40], or PI tethering [18,85]. Furthermore, detection of analytes that interact with transmembrane proteins or membrane-bound antibodies within SLBs is also feasible (Figure 6C).These strategies led to developing biosensing systems employing biomimetic microspheres that sense presence of membrane active agents, lipophilic compounds, ions, or ligand receptor interactions as presented in this section, which first starts by listing current applications that may retain an intact SLB or that disrupt it, followed by a critique of these applications.

### 10.1. Current applications

Current applications have sensed (1) molecules that interact with biomembranes through various modes; (2) ions; and (3) ligand-receptor interactions. Interactions of molecules with biomembranes occurs through different mechanisms including inserting themselves into the bilayer to form pores, partitioning into the hydrophobic or hydrophilic areas of the bilayer based on their affinities, or even initiating interactions that lead to complete disruption of the lipid bilayer. We present examples of applications in which each of these varying modes of interactions was detected under section 10.1.1. Biosensing of ions is presented under section 10.1.2. and biosensing of ligand-receptor interactions is presented under section 10.1.3.

#### 10.1.1. Biosensing of molecules that interact with biomembranes

##### 10.1.1.1. Biosensing of membrane active agents

Sensing of membrane active agents or pore forming proteins was performed by detecting superquenching of polymers on solid microspheres, or detecting release of compounds from porous microspheres, both are presented here followed by presenting other modes of detection.

###### Biosensing of membrane active agents employing superquenching

**Figure 7:**
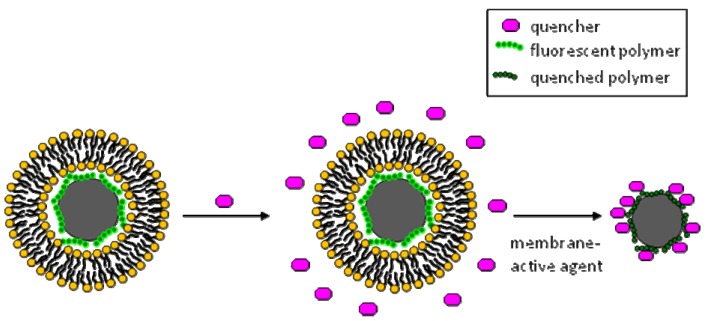
Biosensing of membrane-active agents employing superquenchable polymers. Polymers are deposited on microspheres, then SLBs are formed around them to act as a barrier between the polymer and its quencher. Disrupting the SLB with membrane-active agents permits the close proximity of the quencher and the polymer leading to superquenching.

Employing superquenching with biomimetic microspheres involved adsorbing onto the surface of borosilicate glass microspheres a cationic superquenchable fluorescent polymer, poly(*p*-phynelene-ethynylene (PPE), so that it formed a monolayer (Figure 7) [14,80,86]. Liposomes composed of anionic lipid DMPG were used to coat the polymer-coated microspheres to form lipid bilayers around the microspheres (Figure 7). The lipid bilayer acts as a barrier protecting the fluorescence of the PPE from being superquenched by the anionic quencher anthraquinone disulfonic acid (AQS) [14,80]. Upon introduction of an analyte such as a non-ionic surfactant (Triton X100) or membrane disrupting peptides such as melittin [80], or phopholipase A_2_ (PLA_2_) [86], the lipid bilayer is disrupted, making the PPE available to be quenched by AQS. Alternatively, the SLB was made of AQS-labeled phospholipids and forming it around the PPE-coated microspheres caused their quenching, whereas hydrolysis of the phospholipids released AQS leading to unquenching of PPE [86]. The concentration of the analyte is directly proportional to the measured superquenching, making it a quantitative assay [80]. In addition, combining superquenching with flow cytometry, offers the advantage of increased sensitivity over conventional quenching fluorimetric detection [14]. This format of the assay is a safer sensitive alternative to detecting radioactivity, which was used to quantitate the activity of PLA_2_ with radioactive phospholipids that were coated on styrene divinyl-benzene microspheres [59]. 

###### Biosensing membrane active agents employing release of compounds from biomimetic porous microspheres

Biosensing based on detection of released compounds employs formation of SLBs on porous microspheres encapsulating fluorescent dyes or other chemical agents within the pores of the microsphere. Upon addition of a membrane lytic peptide like melittin or a pore forming protein like α-hemolysin, the SLB is disrupted leading to release of the encapsulated compounds followed by the detection of either their fluorescence or their subsequent interaction with other biomimetic microspheres [45]. This was carried out in suspension and in channels using fluorimetry. 

**Figure 8:**
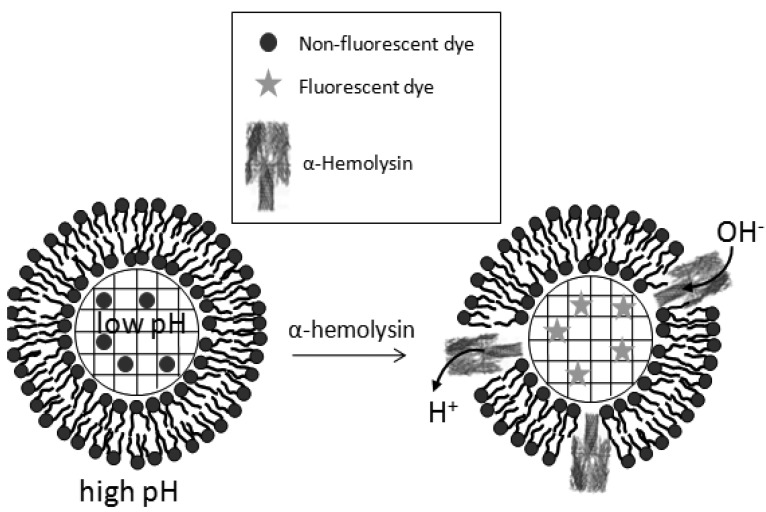
schematic of biosensing of pore-forming α-hemolysin by employing biomimetic porous microspheres enclosing a pH-sensitive fluorescent dye. The outside environment of the microspheres is basic while the inside is acidic which prevents fluorescence of the pH sensitive dye. Adding α-hemolysin forms pores within the SLB allowing ions to pass through the pores, thus increasing the internal pH in the microspheres resulting in detectable fluorescence of the pH sensitive dye.

The assay in suspension involves detection with a fluorimeter or a flow cytometer. Using a fluorimeter required centrifugation of the microspheres after disruption of the SLB to examine the release of the encapsulated dye into the solution [45]. In contrast, using a flow cytometer the microspheres were directly examined and it employed varying the environments inside and outside the biomimetic microspheres (Figure 8). The biomimetic microspheres encapsulated a pH sensitive dye, and the pH inside was acidic while outside it was basic [45]. Adding α-hemolysin caused formation of pores within the SLB which caused exchange of protons and hydroxyl ions across the SLB leading to increased pH inside and thus increased fluorescence (Figure 8). This assay format for α-hemolysin was more sensitive than the alternative suspension assay that examines release of an encapsulated dye into the supernatant after sedimenting the microspheres which is probably a direct outcome of the sensitivity of flow cytometry [45]. 

The assays in channels were designed with either one or two reaction segments. When the encapsulated compound was a fluorescent dye, one reaction segment was used, where the release of the dye was detected below the reaction segment. In contrast, when the encapsulated compound was non-fluorescent, two reaction segments were used. The first reaction segment was the site where membrane active agents reacted with biomimetic microspheres, while the second one involved a fluorescent reaction that was dependent on released agents from the first reaction segment. For example, a channel was designed with two reaction segments, where the first segment contained biomimetic porous microspheres encapsulating non-fluorescent biotin [45]. The biotin was released by disrupting the SLBs to react with the second reaction segment which was separated from the first by a spacer segment of blank microspheres. The second reaction segment contained streptavidin-coated polystyrene microspheres that were reacted with biotin-fluorescein. Reacting non-saturating amounts of biotin-fluorescein with streptavidin causes fluorescence quenching of fluorescein within the second segment, because fluorescein interacts with the *cis* biotin-binding pocket on streptavidin resulting in what is known as ostrich quenching [87]. Releasing biotin from the first segment through SLB disruption displaces biotin-fluorescein in the second segment leading to unquenching of fluorescein, which is detected by fluorimetry [45]. 

##### 10.1.1.2. Biosensing of biomolecule’s affinity to biological membranes employing various modes of detection

There are other examples of membrane-active agents where their affinity to lipid membranes has been carried out with lipid bilayers or monolayers on silica microspheres. For example detecting lipophilicity of drugs was performed using SLBs on microspheres that were referred to as TRANSIL system, which is made of silica microspheres coated noncovalently with egg yolk PC and is commercially available from NIMBUS Biotechnologie GmbH, (Leipzig, Germany). Drugs were incubated with the TRANSIL beads, then phase separation of lipid and aqueous phases were easily done by centrifugation or filtration, and the remaining drug in the aqueous phase was quantified [46]. This assay is fast and is amenable to high throughput screening.

Another example involved evaluation of interactions of peptides (bombesin, β-endorphin, glucagon, and a class A amphipathic peptide) with lipid membranes by employing covalently linked PC monolayers on porous silica microspheres that were packed into a chromatography column. In this case, the retention/elution of peptides was evaluated by monitoring absorbance after flowing through the column varying concentrations of solvents [47,88]. In addition, interactions of myelin basic protein (MBP) with anionic lipids within an SLB on microspheres was detected by NMR and DSC [89]. Furthermore, detection of antibodies to phospholipids have been done using fluorescent techniques on blood of patients with anti-phospholipid syndrome who have increased risk of thrombosis, thrombocytopenia, and fetal loss [1,3]. In that case, cardiolipin or phosphatidyl serine were incorporated into SLB on glass microspheres, and antibodies to them in blood samples were detected by flow cytometry using a fluorescent anti-human antibody [1,3]. All the above are examples of various modes of detecting biomolecule’s affinity to biological membranes.

#### 10.1.2. Biosensing of ions

SLBs on microspheres have been used for quantitative sensing of halide ions, and were referred to as lipobeads [90]. Ma and coworkers [90] described an in suspension assay where polystyrene submicron particles were coated with phospholipid membranes containing a noncovalently immobilized halide-sensing fluorescent dye. The fluorescence of the dye was quenched by halide ions, thus enabling their detection. A newer generation of silica lipobeads was used in a suspension assay to develop pH-based biosensors for the detection of urea [48]. In this study, the sensing lipobeads were prepared by coating carboxyl functionalized silica microspheres with phospholipids. The pH indicator fluorescein-5-thiosemicarbazide and the enzyme urease were then covalently linked to the supported phospholipid membrane. The conversion of urea to ammonia by urease increased the pH of the solution and this led to increased fluorescence of the fluorophore, which was directly proportional to the concentration of the urea present. The increase in fluorescence was detected using FTIR and digital fluorescence-imaging microscopy. Such lipobeads were further used for intracellular pH measurements and intracellular oxygen measurements [91,92].

#### 10.1.3. Biosensing of ligand-receptor interactions

Ligand-receptor interactions were detected using biomimetic microspheres by employing examination of either colloid phase transitions or fluorescence, and both are presented here.

##### 10.1.3.1. Biosensing of ligand-receptor interactions *via* detection of colloid phase transitions

Biosensing of interactions between ligands incorporated into SLBs on microspheres with their receptor that were added to the reaction tube was detected through examination of colloidal phase transitions [5]. For example, interactions of ganglioside G_M1_ with its receptor, cholera toxin was performed using silica microspheres coated with SLBs containing ganglioside G_M1_. Interactions were detected using bright field and fluorescence microscopy where in the absence of the receptor protein (FITC labeled Cholera toxin) the microspheres were in a condensed phase. Adding cholera toxin caused a transition from a condensed colloidal phase to a dispersed colloidal phase within seconds. This transition in phase was also demonstrated to be a highly specific molecular interaction [13].

##### 10.1.3.2. Biosensing of ligand-receptor interactions *via* detection of fluorescence

Biosensing of interactions of biomolecules was done by either incorporating the biomolecules into the SLB or by anchoring biomolecules into SLBs. An example of biosensing of biomolecules within SLBs on microspheres is detection of ICAM-1, a cell adhesion molecule, which was incorporated into SLBs deposited on microspheres, and was detected using a fluorescently-labeled antibody for it [6]. Examples of biosensing interactions with biomolecules anchored onto the SLB included evaluation of binding affinity of drugs to human serum albumin which was done by immobilizing albumin on TRANSIL system. In this case, the SLB reduced nonspecific binding of drugs, and unbound drug molecules were separated by filtration or centrifugation and then quantified [4]. Other examples included detecting biointeractions of streptavidin with biotin-PE within SLBs on microspheres, or even biointeractions of fluorescently-labeled, biotinylated FLAG peptide with streptavidin that is bound to biotin-PE in SLBs on microspheres [6].

### 10.2. Critique of current applications

**Figure 9:**
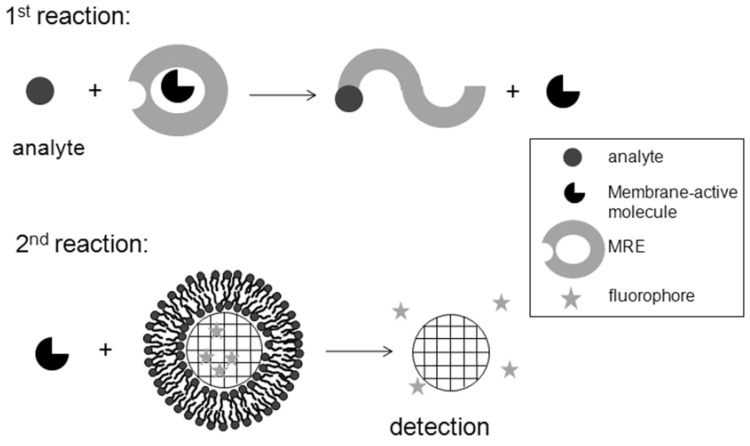
Schematic of a proposed general design that employs biomimetic microspheres for biosensing of analytes that do not interact with biomembranes. This design involves indirect detections with two-step reactions. The first one releases a membrane-active molecule as a result of interaction of the analyte with its MRE. The second reaction detects the released membrane-active molecule after interacting with SLBs on biomimetic microspheres. Fluorimetric detection is shown here as an example.

As listed above biomimetic microspheres have been used in a variety of boisensing applications. It is easy to design biosensors with biomimetic microspheres that detect direct interactions with SLBs such as pore-forming proteins or membrane-disrupting agents. In contrast, the challenge is to design biosensors that can detect presence of molecules or detect interactions between any two molecules that do not necessarily interact with phospholipids within the SLB. The closest to such possible designs was the detection of colloidal phase transition as a result of interaction of molecules in solution with others within the SLBs. Another example that involved indirect biosensing of an analyte through converting it to a product that can be sensed employing a biomimetic microsphere system was described under biosensing of ions (section 10.1.2. above). Although these are two successful examples of biosensing molecules that do not necessarily interact with phospholipids within SLBs, a more general design that can be used with slight modifications is still lacking. Such a design would most likely entail indirect biosensing that involves two reaction steps as proposed in Figure 9. The first reaction step would expose a membrane active agent upon interaction of an analyte with its MRE. This first step may involve either (1) tethering or encapsulating within microspheres a biomembrane active molecule that gets released because of interactions of an analyte with its MRE; or (2) a within solution reaction of an analyte with its MRE leading to exposing or releasing a chemically conjugated biomembrane active molecules. In the second step the released biomembrane active molecule will in turn interact with biosensing biomimetic microspheres present within the mixture or within a second reaction segment in a microchannel. In that case the extent of detected fluorescence will correlate with the amount of present analyte.

The applications mentioned above used either fluorescence or superquenching combined with flow cytometry. Flow cytometry and superquenching, each offers increased sensitivity that is further enhanced when they are both combined together. Nevertheless, other detection techniques have not been explored yet with biomimetic silica microspheres, although some of them have been used with liposomes. For example electrochemical detection with the use of electrodes has been used with liposomes that encapsulate electrochemical compounds such as potassium ferrohexacyanide [64], but has not been evaluated yet with biomimetic microspheres. In addition to potassium ferrohexacyanide, electrochemical nanomaterials may be encapsulated within biomimetic silica microspheres. Other biosensing techniques employed with liposomes, but are not explored yet with biomimetic silica microspheres include QCM and SPR [64]. In these cases the analytes were incorporated into liposomes to enhance detection levels as a result of the liposome size or the bilayer composition [64]. Moreover, magnetic detection is a possibility if nanomaterials with magnetic characteristics are loaded into porous biomimetic microspheres. Even higher fluorescence sensitivity and stability to photobleaching is achievable by loading porous biomimetic microspheres with quantum dots (QDs) which have high quantum yields. In fact, in addition to forming lipid layers on microspheres, they have been formed on nanospheres such as hydrophobic nanocrystals, or QDs [93]. Additionally, instead of supporting liposomes on nanospheres, they have been loaded with monodisperse magnetic nanocrystals [94,95]. However, this was not covered here as it is outside the scope of this review which focuses on biomimetic silica microspheres. Loading of biomimetic microspheres with nanomaterials with various characteristic will introduce new detection methods. One last critique is that so far the current applications employed SLBs made of phospholipids without incorporating any cell-derived fragments into them, although it is possible to incorporate such fragments as presented above under section 4.2. and as discussed further below under conclusions. 

## 11. Conclusions

This review presented biomimetic microspheres composed of SLBs deposited on silica microspheres that are used for biosensing applications. The use of microspheres in biosensing is appealing because of their increased detection limit as a result of increased concentration of bound analyte on the surface of the microspheres relative to the bulk solution, and possibilities for miniaturization, multiplexing, and high throughput screening.

The most popular biomimetic materials used on microspheres are lipid mono- or bi-layers supported on microspheres. Biomimetic lipid layers allow the incorporation of transmembrane proteins or tethering of biomolecules for appropriate biosensing applications. Lipid supported microspheres have been used in suspension, or packed in channels for biosensing applications and it is possible to use them within chemically etched wells on optical fiber bundles (Figure 5). All these formats utilize for detection the fluorescence properties of molecules incorporated into the biomimetic microsphere, and all are amenable for high throughput screening of analytes.

We presented various biomimetic materials that have been used, or can be used with biomimetic microspheres including liposomes and cell-derived vesicles. Other unexplored materials that can be used as biomimetic microspheres either without modifications or after incorporating with liposomes include bacterial ghosts, which are bacterial cell envelopes prepared from gram negative bacteria [96] and Attoliter vesicles [97]. Attoliter vesicles are cell fragments made of membranes and some cytosolic components [97]. They are prepared by treating the cells with the drug cytochalasin B, which rapidly disrupts the actin cytoskeleton. Gentle agitation of the cells leads to formation of monodisperse vesicles, which are referred to as Attoliter vesicles [97]. Varying the dosage of the drug or the extent of agitation of the cells controls the size of the formed vesicles. The size of the vesicles has been found to range from a few hundred nanometers to a few micrometers in diameter. These vesicles have been found to retain their functional integrity even after being stored for many weeks [97]. Although Attoliter vesicles are biomimetic microspheres, they can be combined with hard components for additional biosensing uses. Attoliter vesicles could be used to coat porous microspheres with or without initial incorporation into liposomes. This could facilitate sensing changes in cell signaling at the cytoplasmic side of the vesicles especially when specific cytoplasmic molecules are encapsulated within the porous microsphere. 

We described under section 4 the use of erythrocytes ghost membranes that are prepared by reducing the osmotic pressure [42]. These are currently tested for drug delivery [98], and when used with the same patient that they were collected from, that gives the advantage of their biocompatibility with their host. These ghosts can be easily modified to serve as *in vivo* biosensors in the blood stream, also with matching the donor to the host. For example, they can be transfected with particular receptors that can serve as MREs on their cell surfaces and be modified or loaded with a transducer for the purpose of being injected into the blood stream to detect molecules or pathogens in the blood.

Biomimetic microspheres can be easily adapted for multiplexed assays because of their high surface area, and ease of use in flow cytometry, which is already adapted for multiplexing and encoding. So far, research has focused on silica biomimetic microspheres, and several permutations of materials and biomolecules have not been tested with biomimetic microspheres. For example, microspheres with metallic cores, or porous microspheres encapsulating chromophoric nanocrystals, quantum dots, magnetic nanomaterials, or encapsulating biomolecules that retain their functions can be employed. This will add to the variety of designs of biosensors, improve detection methods, and permit detection of events involved in cellular signal transduction pathways. Finally future designs on biomimetic microspheres will be robust and involve compartmentalization within microspheres for multi-analyte detection or particular designs that allow complementation of reactions within different compartments contained by one microsphere. Such designs mimic the compartmentalization inside live cells, and allow for detection of complex reactions.
